# Predicting double negativity using transmitted phase in space coiling metamaterials

**DOI:** 10.1098/rsos.171042

**Published:** 2018-05-16

**Authors:** Santosh K. Maurya, Abhishek Pandey, Shobha Shukla, Sumit Saxena

**Affiliations:** Nanostructures Engineering and Modeling Laboratory, Department of Metallurgical Engineering and Materials Science, Indian Institute of Technology Bombay, Mumbai, Maharashtra 400076, India

**Keywords:** negative refractive index, metamaterials, acoustics

## Abstract

Metamaterials are engineered materials that offer the flexibility to manipulate the incident waves leading to exotic applications such as cloaking, extraordinary transmission, sub-wavelength imaging and negative refraction. These concepts have largely been explored in the context of electromagnetic waves. Acoustic metamaterials, similar to their optical counterparts, demonstrate anomalous effective elastic properties. Recent developments have shown that coiling up the propagation path of acoustic wave results in effective elastic response of the metamaterial beyond the natural response of its constituent materials. The effective response of metamaterials is generally evaluated using the ‘S’ parameter retrieval method based on amplitude of the waves. The phase of acoustic waves contains information of wave pressure and particle velocity. Here, we show using finite-element methods that phase reversal of transmitted waves may be used to predict extreme acoustic properties in space coiling metamaterials. This change is the difference in the phase of the transmitted wave with respect to the incident wave. This method is simpler when compared with the more rigorous ‘S’ parameter retrieval method. The inferences drawn using this method have been verified experimentally for labyrinthine metamaterials by showing negative refraction for the predicted band of frequencies.

## 1. Introduction

Metamaterials are a class of artificially engineered materials which offer the possibility of achieving effective properties beyond the scope of naturally existing properties of constituent elements. These are envisioned to have several important applications such as surface wave manipulation, sub-wavelength focusing [1–5], extraordinary transmission [6–9] and cloaking [10,11]. While early developments can be traced back to 1967 in the theoretical report by Veselago [12], the first experimental realization of such materials was reported only in 2001 [13] by simultaneously achieving negative permittivity and negative permeability using a split ring resonator. The concept of metamaterials was initially developed with an intent to manipulate electromagnetic waves.

This idea of electromagnetic wave manipulation using metamaterials was soon adapted and extended to manipulate acoustic wave propagation. This enabled achieving energy flow in the direction opposite to that of sound propagation. Sound wave propagation can be controlled by mass density and bulk modulus of the material, which are both positive in conventional materials. One of the first demonstrations of acoustic metamaterials [14] used locally resonant phenomenon to show peculiar acoustic properties. These were followed by the development of sub-wavelength-sized Helmholtz resonator-based designs [15]. Subsequently, other resonant elements, such as transmission line networks [16] and tensioned membranes [17], were also used. Space coiling is a recently proposed phenomenon in the area of acoustic metamaterials [18,19]. These do not use the traditional concept of local resonance. In this approach, sound waves are forced to propagate through passes that are much longer than their external dimensions. This enables a low refractive index material to mimic as an effective medium with very high refractive index. The effective index and the dispersion relation can be tuned by adjusting the total length of the path of the low refractive index medium. Space coiling structures are capable of generating abrupt phase shifts which provide additional momentum to the incident wave, thereby causing it to behave abnormally. Several approaches to coil up space in two and three dimensions have been proposed [20,21]. These developments in the area of acoustic metamaterials have resulted in a novel approach to achieve sound attenuation, steering of waves, one-way sound transport [22], nonlinear elastic wave sensing [23] etc.

The properties of the metamaterials can be evaluated using several methods based on measurements or simulations of reflection and transmission coefficients. The ‘S’ parameter retrieval method was proposed by Nicolson and Ross in 1970 [24] and is the most commonly used technique to calculate the properties of metamaterials. This technique was further developed by Smith *et al*. [25] for electromagnetic waves and later adapted for acoustic waves. In this method, the effective refractive index and the impedance are obtained from complex reflection and transmission coefficients also known as ‘S’ parameters through complicated expression. Another technique based on analysis of the acoustic band dispersion is based on the use of the Floquet–Bloch theorem. This requires construction of an equivalent cell which may be fairly complicated to design especially for three-dimensional (3D) space coiling acoustic metamaterials. Thus, a simplistic approach is required which enables in quick estimation of anomalous properties of metamaterials. Determining phase of the transmitted wave is relatively simpler. Here, we analyse phase of the transmitted acoustic wave using finite-element modelling to estimate the anomalous behaviour of two-dimensional (2D) and 3D labyrinthine structures. The findings from phase analysis of transmitted waves are in agreement with those obtained from the rigorous ‘S’ parameter retrieval method and acoustic band analysis using the Floquet–Bloch theorem. Furthermore, experimentally determined acoustic pressure measurements show negative refraction of acoustic waves in the acoustic bands predicted by analysing the phase of the transmitted wave. The method of calculation of change in the phase of transmitted wave may be used as a marker for predicting acoustic band with negative refractive index.

## 2. Material and methods

Finite-element method was used to simulate 2D and 3D labyrinthine structures in a rectangular acoustic waveguide to obtain the transmission wave properties. These calculations were also used to calculate the ‘S’ parameters to determine the refractive index and acoustic impedance explicitly. Material properties were chosen to be isotropic. The equivalent structure was made of aluminium with channels containing hypothetical material with an effective high refractive index. The density of aluminium was taken to be 2700 kg m^–3^ and speed of sound as 6420 m s^–1^. The density for hypothetical high refractive index medium was taken to be approximately 84 kg m^−3^ and the speed of sound in this medium was taken to be approximately 42 m s^–1^. Inhomogeneous Helmholtz equation was used as acoustic wave equation. Plane wave with a pressure amplitude of 1 Pa was used as the incident wave. Periodic boundary conditions are applied normal in the direction of propagation of the wave. Sound soft conditions were used to avoid internal reflections.

**Figure 1.**
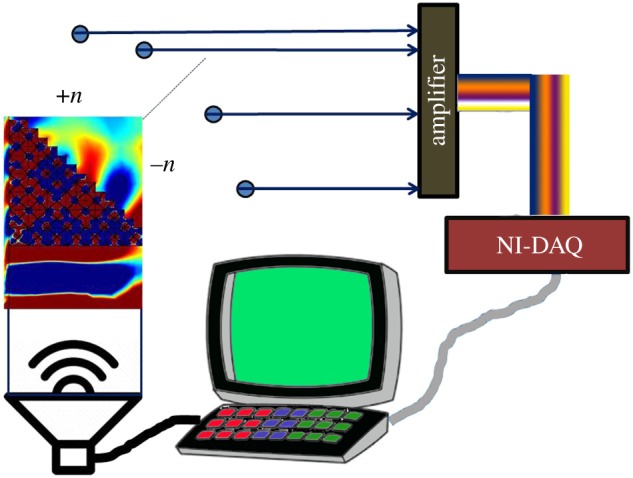
Schematic of the experimental set-up used for the demonstration of negative refraction for 2D and 3D labyrinthine structures. The blue dots are representatives of microphones arranged in an array interfaced to a DAQ card via amplifiers.

Experiments were performed to observe negative refraction in the predicted band of frequencies. The 2D and 3D labyrinthine unit cells were fabricated and arranged in a prismatic arrangement. Sound was generated using a 5 Ω speaker. An array of microphones were arranged at equal separation (radially) from the prismatic arrangement of metamaterial unit as shown in figure 1. The microphones were interfaced via a custom-made amplifier to a NI-DAQ (4351). The signal was acquired using LabVIEW. The transmission measurements were performed by stacking several 3D units as a wall inside a waveguide with source at one end and a detector at the other. Further details of transmission measurements have been described elsewhere [26].

## 3. Results and discussion

Phase of a longitudinal wave contains information about its wave pressure and particle velocity. Thus, it can be used to engineer the wave propagation characteristics by modifying the geometry of the medium through which the wave traverses. To understand the correlation of the transmitted wave with extreme properties, the transmitted wave characteristics have been investigated for 2D and 3D labyrinthine structures.

**Figure 2.**
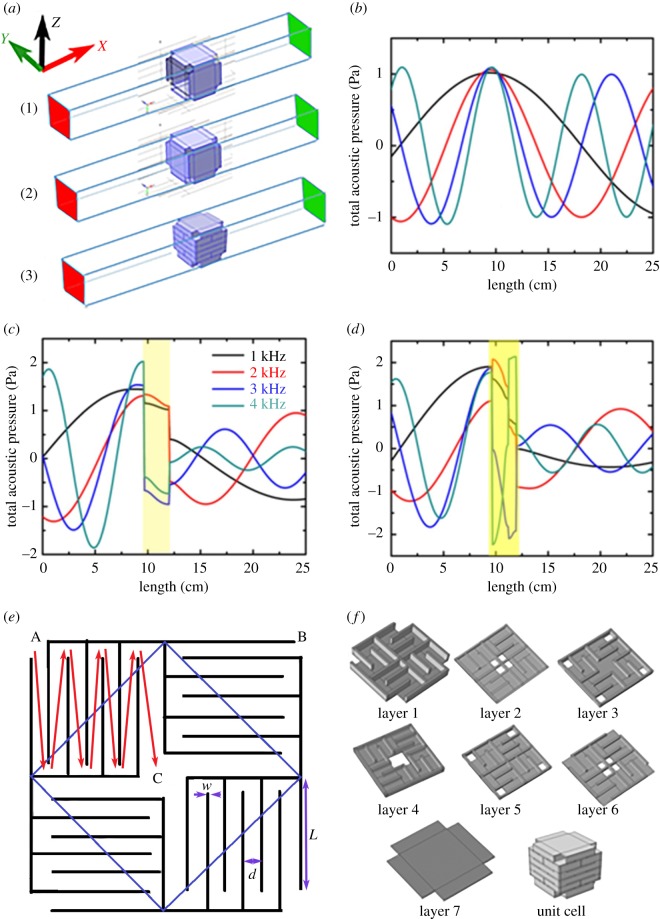
(*a*) Schematic of simulation set-up for determining the transmission properties. Panel (1) shows the surrounding shell, panel (2) shows the complete outer shell, while panel (3) shows the meta unit inside a waveguide. The source is placed at left (red) end, while the detector is placed at the right (green) end of the waveguide. Transmitted wave characteristics for (*b*) outer surrounding shell (excluding the front and rear surface), (*c*) complete outer shell and (*d*) single meta unit in the waveguide. Schematic of unit cell for (*e*) 2D (*w* = 0.8 mm, *d* = 1.5 mm, *L* = 10 mm) and (*f*) 3D labyrinthine space coiling structures.

Sine waves with frequencies in the range of 1 kHz to 4 kHz were incident from the left end of the waveguide on the unit cell placed inside the waveguide as shown in figure 2*a*. A monitor was placed at the right end of the waveguide to determine the transmitted wave characteristics. Transmitted wave characteristics for the surrounding shell (panel 1 in figure 2*a*), the complete outer shell (panel 2 in figure 2*a*) and the meta unit (panel 3 in figure 2*a*) are shown in figure 2*b–d*, respectively. Figure 2*b* shows that the surrounding shells participate insignificantly in reflection of the incident wave. Amplitude of the wave remains almost unchanged and the wave gets transmitted almost without attenuation as expected. However, as soon as the front and rear surfaces are incorporated, a significant decrease in amplitude of the transmitted wave is observed as in figure 2*c*. This happens due to the impedance mismatch at the interface. The inclusion of space coiling structure introduces many such interfaces and transmitted wave develops an overall phase difference while exiting the meta unit as seen in figure 2*d*. The schematic cross sections of the 2D and 3D labyrinthine structures are shown in figure 2*e*, *f*, respectively. Transmitted wave profiles obtained were fitted with equation of sine wave to determine the phase difference developed during the transition. The relative phase of the transmitted wave was obtained by taking the difference of the phase of the incident wave from that of the transmitted wave.

**Figure 3.**
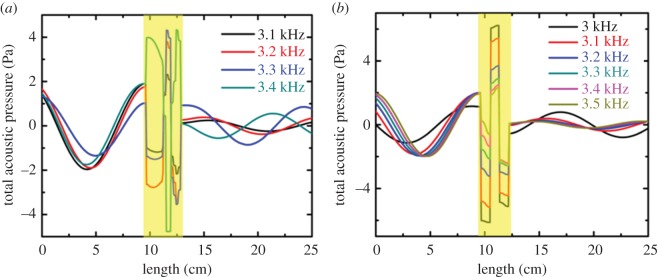
Transmission wave characteristics for (*a*) 2D maze and (*b*) 3D labyrinthine space coiling metamaterials for different frequencies. The positions of the metamaterial unit in the simulation box are represented by yellow region.

The change in phase of wave depends on the frequency as seen in figure 3*a*,*b*. Amplitude of the total acoustic pressure changes from positive to negative as seen in figure 3*a* at approximately 3.4 kHz for 2D maze.

**Figure 4.**
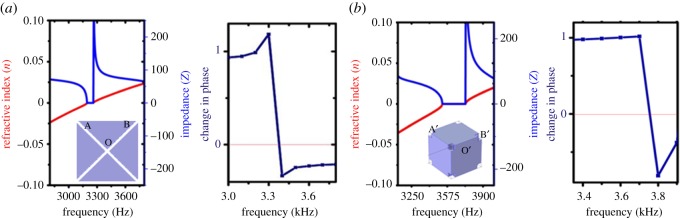
Effective refractive index (*n*), impedance (*Z*) and change in phase obtained for (*a*) 2D maze and (*b*) 3D labyrinthine space coiling metamaterials. Refractive index and impedance have been obtained using the ‘S’ parameter retrieval method. The inset shows equivalent cells for 2D and 3D structures. AOB (in (*a*) inset) represents the effective path travelled by the acoustic waves in 2D maze, while A'OB' (in (*b*) inset) represents analogous path in 3D equivalent cell.

The slope of the change of phase turns negative at approximately 3.3 kHz for the 2D and approximately 3.7 kHz for the 3D structures as seen in figure 4*a*,*b*. This change of phase of transmitted wave remains negative suggesting anomalous behaviour of acoustic wave propagation. The propagation of acoustic waves in space coiling labyrinthine structures resembles motion of ultraslow fluid particles compared to the background. This enables extension of Mie theory to understand the anomalous behaviour in space coiling structures for acoustic waves. This extension to acoustic waves relates monopolar Mie resonance to the negative bulk modulus, while the dipolar resonances to negative mass density [27].

The pressure difference force is related to the pressure gradient. The change in phase from positive to negative would correspond to negative density gradient and hence this change in phase is expected to act as a marker for the negative properties. Similar observations have been made in the case of other metamaterials [28] and optical counterparts of acoustic metamaterials [29]. The advancement of negative phase [30] has also been understood to bend the light in negative direction at the exit interface in optical metamaterials [31]. The space coiling structures show double negativity at frequency near the band folding position for the same frequency range as obtained from phase analysis and the ‘S’ parameter retrieval method.

To calculate the effective refractive index of the metamaterial structures, equivalent cells as shown in figure 4*a*,*b* were constructed. The path AOB (inset in figure 4*a*) represents the path of acoustic waves travelling through the medium in the 2D structures. A'OB' (inset in figure 4*b*) represents similar acoustic wave path in the case of 3D structures. The bluish region represents the material used for the construction of metamaterial structures. The refractive index of the white region, which is a hypothetical high refractive index material, provides a measure of coiling up of space in the space coiling structure. This is obtained by dividing the total path length in the actual structure by the path length in the equivalent cell. The refractive index (*n*) and impedance (*Z*) can be expressed in terms of the reflection (*S*_11_) and transmission (*S*_21_) coefficients by the following relations:

$$n=\frac{\pm \cos^{-1}[(1-S_{11}^{2}+S_{21}^{2})\text{/}2S_{21}]}{kd}$$

and

$$Z=\pm \sqrt{\frac{{\left(1+S_{11}^{2}\right)}^{2}-S_{21}^{2}}{{\left(1-S_{11}^{2}\right)}^{2}-S_{21}^{2}}}.$$

These have been plotted in figure 4*a* for 2D maze structure. Similar calculations were performed for 3D structures and have been plotted in figure 4*b*.

**Figure 5.**
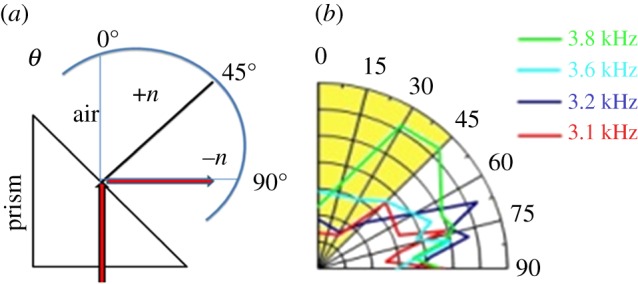
(*a*) Schematic showing the positive and negative refractive index region of transmitted wave in prismatic arrangement of 3D meta units. (*b*) Polar plot of normalized transmitted wave amplitude when the wave travels through the prismatic arrangement of meta units. The radial direction represents normalized transmitted amplitude.

As a final check, experiments were performed to detect the refracted wave in the negative refractive index regime of a prismatic arrangement of 2D and 3D space coiling structures as shown in figure 5*a* in the range of frequencies predicted by transmitted phase analysis.

Total acoustic pressure was measured at various angles using an array of condenser microphones and a polar plot was constructed by representing the radial direction as the amplitude of the transmitted acoustic pressure. It is observed that at approximately 3.8 kHz in figure 5*b*, the transmitted wave amplitude lies predominantly in the positive refractive index region, while for all frequencies below down to 3.1 kHz, the transmitted waves propagate predominantly in the region of negative refractive index. Thus the polar plot provides a direct experimental verification of negative refraction in the range of frequencies as predicted by the phase analysis of transmitted wave.

## 4. Conclusion

Finite-element methods have been used to calculate the transmitted waves profile of acoustic waves passing through 2D and 3D space coiling metamaterials. The analysis of the transmitted waves shows that phase is developed while traversing through the metamaterial unit. This may be used to predict negative refraction in space coiling structures. This approach reduces the computational complexity for estimating extreme properties when compared with the ‘S’ parameter retrieval method. Our investigation suggests that the phase of the transmitted wave and hence the phase velocity undergoes a reversal while travelling through the space coiling structures when compared with normal propagation of acoustic waves. Experimental data show negative refraction of sound waves in the range of frequencies predicted using phase analysis of transmitted wave. To conclude, the change in the phase of the transmitted wave can be used as a marker for qualitatively predicting negative refraction in space coiling structures.

## Supplementary Material

3D experimenal data.opj

## Supplementary Material

fig3a_data.txt

## Supplementary Material

fig3b.txt

## Supplementary Material

Outer_shell.txt

## Supplementary Material

transmission_3D.txt

## Supplementary Material

transmission_outer.txt

## Data accessibility

All data are available in the electronic supplementary material.
